# In vivo optical imaging of early osteoarthritis using an antibody specific to damaged arthritic cartilage

**DOI:** 10.1186/s13075-015-0898-5

**Published:** 2015-12-25

**Authors:** Ngee Han Lim, Tonia L. Vincent, Ahuva Nissim

**Affiliations:** Centre for Biochemical Pharmacology, William Harvey Research Institute, Barts and The London School of Medicine and Dentistry, Queen Mary University of London, London, EC1M 6BQ UK; Arthritis Research UK Centre for OA Pathogenesis, Kennedy Institute of Rheumatology, NDORMS, University of Oxford, Roosevelt Drive, Headington, Oxford, OX3 7FY UK

**Keywords:** Osteoarthritis, Antibody, Imaging, DMM, Reactive oxidants

## Abstract

**Background:**

The lack of specific and sensitive serum and radiographic biomarkers for early diagnosis of osteoarthritis (OA) as well as for monitoring subtle changes in disease activity in clinical trials has hampered the development of treatments for OA. We previously showed that 1-11E, a human single chain fragment variable (scFv) specific to collagen type II that has been post-translationally modified by reactive oxidants (ROS-CII), binds exclusively to arthritic cartilage. Here we test the validity of 1-11E as a radiographic biomarker for early disease in experimental OA.

**Methods:**

Murine OA was induced by destabilisation of the medial meniscus (DMM) in adult male mice. Immunohistochemistry of destabilised or sham-operated knees was performed from 2 to 8 weeks post-surgery with Cy5.5-labelled 1-11E and negative control scFv, C7. Prospective in vivo optical images were taken 4 and 8 weeks post-DMM following intra-articular injection of Cy5.5-labelled scFvs, or intravenous injection of Cy5.5-labelled full length monoclonal antibodies (mAbs).

**Results:**

Specific cartilage staining with 1-11E was apparent as early as 4 weeks post-DMM at the time of earlier cartilage degradation assessed by histology. Prospective in vivo optical images taken 4 and 8 weeks post-DMM following local intra-articular injection of Cy5.5-labelled scFv (n = 7) showed specific in vivo retention of Cys5.5-1-11E scFv following local administration into the knee joint (tissue half-life >78 hours, n = 7, signal to noise ratio (SNR) > 2.1). Specific localization of Cys-5.5-1-11E-mAb to DMM knees (SNR >1.65) was also observed (*p* < 0.01, n = 8, SNR >1.65). In both cases the SNR increased with time post-DMM.

**Conclusions:**

1-11E binds specifically to early osteoarthritic cartilage and can be used as a radiographic biomarker following local or systemic delivery to facilitate early diagnosis and monitor disease progression in OA.

## Background

Osteoarthritis (OA) is a disease of the whole joint with articular cartilage breakdown as a major characteristic, but also involving pathology within the synovium, bone, menisci, ligaments, muscles and neural tissues [1, 2]. OA is the most common joint disease, with a population-wide prevalence of symptomatic disease of approximately 15 %, 12 % and 6 % in the hand, knee and hip, respectively [3]. Ageing of the world’s population is likely to increase the burden of this disease further [4].

Given the huge economic and personal burden of OA, there is an urgent unmet need to develop disease-modifying OA drugs (DMOADs) that can reduce or stop its progression. Treatment with drugs such as non-steroidal anti-inflammatory drugs, opioid-derived analgesic drugs or locally administered corticosteroids have moderate effects on symptomatic disease, but for many patients joint replacement surgery represents the only hope of relief [5]. The introduction of potential DMOADs, however, has been hampered by the lack of specific and sensitive biomarkers capable of detecting early disease or discerning modest changes in disease progression. Much funding has gone into the search for novel serum, urine and synovial fluid biomarkers of disease progression, but thus far no single soluble biomarker has been shown to have value either in disease severity prediction or progression within an individual [6, 7]. Of these markers, only serum cartilage oligomeric protein levels maintained association with OA in large-scale studies with a low odds ratio of 3.26 [8].

The current gold standard for disease evaluation, in spite of its limitations, is the plain radiograph, which relies on the presence of relatively late features of the disease, including presence of osteophytes, joint space narrowing (signifying marked cartilage loss) and bone remodeling [9]. X-ray images are insensitive to early changes within the joint and do not report on cartilage or synovial pathology as these soft tissues are transparent to X-rays. Although magnetic resonance imaging (MRI) is a more sensitive and specific radiographic tool for assessment of OA joint changes (including cartilage loss, synovitis, bone oedema, etc.) [9], its widespread use in clinical practice is hampered by cost, long acquisition times and poor patient acceptability [10]. MRI is, however, becoming a key imaging tool for OA research [9, 11–15] due to its ability to detect changes at pre-radiographic OA [9, 16]. Another drawback of MRI is that the significance of many MRI features in pre-radiographic OA are still unclear and therefore of limited clinical utility [17].

Biochemical markers in combination or used in conjunction with imaging may prove to be more powerful in establishing stage of disease, predicting progression, and assessing improvement in clinical trials [18]. Potential DMOADs need to be first validated in preclinical studies mostly utilizing small animal models of OA [3, 19, 20]. Currently, disease is assessed by serial histology of the joint which is time consuming, costly and requires large number of animals as they need to be culled at each experimental time point under investigation. Powerful non-invasive preclinical imaging techniques for longitudinal studies are therefore highly desirable for preclinical validation studies as well as for detection and monitoring of early OA in patients.

We have developed a panel of human single chain fragment variables (scFvs) specific for collagen type II that has been post-translationally modified by reactive oxidant species (ROS-CII), known to be present in the arthritic joint [21]. One scFv candidate, namely 1-11E, binds specifically to arthritic cartilage from human OA and rheumatoid arthritis, as well as experimental OA and inflammatory arthritis, but not to healthy cartilage [21, 22]. Hence, 1-11E targeted a payload drug to arthritic joints following systemic administration in inflammatory mouse model of arthritis [21, 22]. Here, we assess the utility of Cy5.5-labelled 1-11E-scFv and monoclonal antibody (mAb) as an imaging probe to detect early disease in mice with experimental OA induced by destabilisation of the medial meniscus (DMM).

## Methods

### Antibody preparation and labelling

1-11E-scFv and control anti-hen egg lysozyme C7-scFv were expressed in HB2151 bacteria as described [23]. Briefly, following induction by isopropyl β-D-thiogalactoside the scFv was harvested from both periplasm and bacterial supernatant followed by purification using protein A Sepharose CL-4B (GE Healthcare, Buckinghamshire, UK) as described [23]. ScFv was converted to full length antibody by cloning V_H_ domain into pFUSEss-CHIg-hG1e3, and V_L_ domain into pFUSEss-CLIg-hk (InvivoGen, San Diego, CA, USA). Following transient expression in HEK-293 F cells, supernatants were collected and purified using protein A Sepharose CL-4B.

Purified 1-11E-scFvs and the control C7-scFvs and their respective mAbs were labelled with Cy5.5 according to the manufacturer’s instructions (GE Healthcare, Buckinghamshire, UK), resulting in a dye to protein ratio of 2.2 and 1.8 for scFv and mAb, respectively. Before injecting the tagged fusion protein to mice, its ability to retain specific binding to ROS-CII over native CII was assessed by enzyme-linked immunosorbent assay as described [21].

### Mouse model of OA

Male C57 BL/6J mice aged 10 weeks were purchased from Harlan Laboratories (Blackthorn, Bicester, UK). Mice were housed in groups of five in individually vented cages, maintained at 21 ± 2 °C on a 12-hour light/dark cycle and with food and water provided. All experimental protocols were performed in compliance with the UK Animals (Scientific Procedures) Act 1986 regulations for the handling and use of laboratory animals (Home Office project licence PPL no: 30/3129).

OA was induced by surgical destabilization of the medial meniscus as described [24]. Briefly, mice were anaesthesised with 3 % isofluorane. Following capsulotomy, the right meniscotibial ligament was transacted, resulting in the release of the medial meniscus from its tibial attachment. Sham surgery consisted of capsulotomy alone. For pain relief, animals were given Vetergesic by subcutaneous injection prior to recovery from anaesthesia. Animals were monitored daily post-surgery.

### Cartilage immunohistochemistry

Serial sections from previously DMM-operated C57BL/6 mice were stained with safranin O according to standard protocols [25]. Cartilage immunostaining was performed on 5 μm thick sections, which were deparaffinised, hydrated, antigen retrieved and blocked as previously described [22]. Sections were incubated overnight at 4 °C with Cy5.5-1-11E-scFv or control Cy5.5-C7-scFv (10 μg/ml) in DAKO antibody diluent solution (Dako, Cambridge, UK). The pericellular matrix was stained using the rat anti-heparan sulphate proteoglycan antibody (Millipore, Watford, UK) followed by the Alexa-Fluor-488 labelled anti-rat IgG (Life Technologies, Paisley, UK). Slides were viewed under the LSM 510 Meta (Zeiss, Cambridge, UK) using the 488 nm excitation laser to visualize the Alexa-488 label and the 633 nm excitation laser to visualize the Cy5.5-label.

### In vivo localisation of 1-11E following DMM surgery

Longitudinal imaging was performed on mice 4 weeks and 8 weeks post-DMM surgery following 1 μg intra-articular (i.a) injection of Cy5.5-labelled scFv or by intravenous (i.v.) injection of 10 μg Cy-5.5-labelled mAb. The knee area of all mice was shaved before fluorescence imaging with IVIS Spectrum imager using the excitation wavelength of 675 nm and an emission wavelength of 720 nm (Perkin Elmer, Waltham, Massachusetts, USA) together with a black and white photograph for orientation. Images were taken at different time points up to 336 hours post-i.a. injection or 48 hours post-i.v. injection. Images were analysed using Living Image 4.4 (Perkin Elmer, Waltham, Massachusetts, USA) to obtain the average fluorescence intensities of a circular region of interest encompassing the knee joint. No background intensities were subtracted from the values; auto-fluorescence is only present in C57BL/6 mice in shaved areas and the data from the contralateral knee joints is shown in all figures and time points to indicate the non-specific retention of antibody. Following the last imaging time point, mice were euthanised by cervical dislocation.

### Statistics and curve fitting

The half-life of the scFv following i.a. injection was calculated using a single-phase decay model with the Prism 6.0 (Graphpad Software, La Jolla, USA). Two-way analysis of variance with Tukey post-tests were performed with Prism software. Permutation analyses with the Wilcoxon rank-sum test were used to compare results across all time points to test whether the DMM operated knee significantly differed from all control groups.

## Results

### Immunohistochemical localisation of 1-11E in OA cartilage

**Fig. 1:**
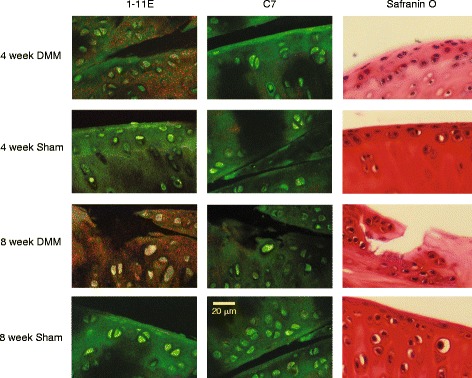
Immunohistochemistry of destabilised medial meniscus (*DMM*) sections at 4 and 8 weeks after surgery. Paraffin sections of knee joints 4 and 8 weeks after DMM surgery were probed with Cy5.5-1-11E or control Cy5.5-C7 scFvs and visualised under the confocal microscope. The Cy5.5 label is false coloured in *red*, and the anti-heparan sulphate proteoglycan antibody detected with Alexa-488 labelled secondary antibody coloured in *green*. The consecutive section was counterstained with Safranin O. Scale bar = 20 μm

Joint sections were probed with the Cy5.5-1-11E-scFv or control Cy5.5-C7-scFv and visualised under the confocal microscope. Staining was observed as early as 4 weeks post-DMM in the area displaying aberrant safranin O staining. No staining was noted with either control C7-scFv or in the sham-operated knee 4 weeks post-operation. Staining of cartilage 8 weeks post-DMM became more evident and spread to deeper layers of the cartilage. Strong staining was seen in the areas of eroded cartilage exhibiting features of active OA with altered Safranin O staining: cell clustering, and clefts (Fig. 1).

### In vivo optical imaging of preclinical OA following intra-articular injection of Cy5.5-1-11E-scFv 4 and 8 weeks post-DMM

We next carried out i.a. injection of Cy5.5-1-11E-scFv into the OA joint to test the local joint retention of Cy5-1-11E-scFv. Mice were injected i.a. with 1 μg Cy5.5-1-11E-scFv (n = 7) or control Cy5.5-C7-scFv (n = 5) 4 and 8 weeks post-surgery. Clearance of Cy5.5-1-11E-scFv or control Cy5.5-C7-scFv was followed using in vivo optical imaging over 336 hours (2 weeks). The amount of scFv detected at each time point was expressed as the percentage remaining compared with the amount present in the joint immediately after injection (time zero), which was taken as 100 %.

**Fig. 2:**
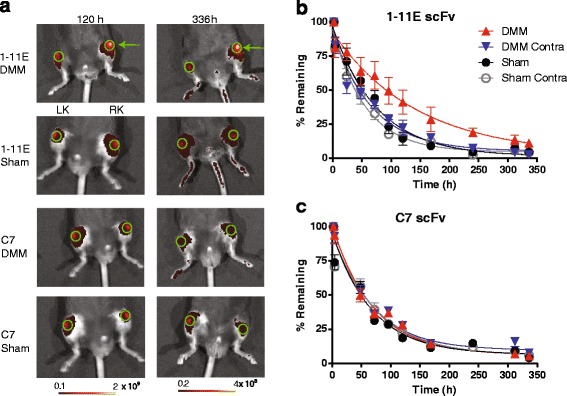
Retention of Cy5.5-1-11E-scFv in mice following i.a. injection 4 weeks post-DMM surgery. **a** Representative fluorescent overlay images of mice 4 weeks post-surgery and 120 and 336 hours after i.a injection of 1 μg Cy5.5-1-11E-scFv or Cy5.5-C7-scFv. *Green* circles denote the regions of interest used for quantification; *green arrow* indicates the right DMM-operated knee (*RK*); *LK* is the non-operated contralateral knee. The amount of injected Cy5.5-1-11E-scFv (**b**) and Cy5.5-C7-scFv (**c**) remaining in the knee joints are expressed as a percentage with 100 % taken as the amount present immediately after i.a. (time 0) injection. Data are mean ± SEM, n = 7 for 1-11E-scFv injected group and n = 5 for C7-scFv injected group. *p < 0.05 between the half life of 1-11E-scFv in the DMM vs the other groups. *DMM* destabilised medial meniscus-operated knee, *DMM Contra* non-operated contralateral knee, *scFv* single chain fragment variable, *Sham* sham-operated RK, *Sham Contra* non-operated contralateral knee

At 4 weeks post-DMM there was no significant difference observed between the amounts of Cy5.5-1-11E-scFv DMM in the knee versus the contralateral knee or sham-operated knee until 120 hours (5 days) post-i.a. injection (41.1 ± 8.6 % Cy5.5-1-11E-scFv remained in the DMM-operated joints, 22.3 ± 5.7 % in the contralateral knee and 14.5 ± 4.7 % in sham-operated joints), after which there was higher signal in the DMM knee compared with the contralateral knee and control sham-operated groups (Fig. 2a). There was significantly reduced clearance of Cy5.5-1-11E-scFv from DMM-operated knees, with a mean of 11.15 % 1-11E scFv remaining 336 hours after i.a. injection, compared with 2.58 % remaining in the contralateral knee and 2.37 % remaining in the sham-operated knee (Fig. 2b; n = 7, *p* = 0.01). The half-life of Cy5.5-1-11E-scFv in the DMM-operated knee was 78 hours, which was significantly (*p* < 0.05) longer than the DMM contralateral (40 hours) and the sham-operated (50 hours) knees. No significant difference in the clearance was observed in DMM knees injected with the control Cy5.5-C7-scFv compared to either the contralateral or sham-operated knees (Fig. 2c; n = 5, *p* = 0.334). The half-life of Cy5.5-C7-scFv in the DMM-operated knee was 50 hours which was not significantly different from the DMM contralateral (47 hours) or the sham-operated (44 hours) knees.

**Fig. 3:**
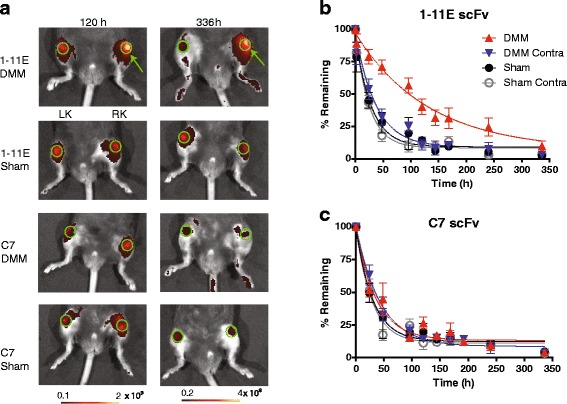
Retention of Cy5.5-1-11E scFv in mice following i.a. injection 8 week post-DMM surgery. **a** Representative fluorescent overlay images of mice 8 weeks post-surgery and after 120 and 336 hours i.a injection of 1 μg Cy5.5-1-11E-scFv or Cy5.5-C7-scFv. *Green* circles denote the regions of interest used for quantification; *green arrow* indicates the right DMM-operated knee (*RK*); *LK* is the non-operated contralateral knee. The amount of injected Cy5.5-1-11E-scFv (**b**) and Cy5.5-C7-scFv (**c**) remaining in the knee joints are expressed as a percentage with 100 % taken as the amount present immediately after i.a. (time 0) injection. Data are mean ± SEM, n = 7 for 1-11E-injected group and n = 5 for C7-injected group. *p < 0.05 between the half life of 1-11E-scFv in the DMM vs the other groups. *DMM* destabilised medial meniscus-operated knee, *DMM Contra* non-operated contralateral knee, *scFv* single chain fragment variable, *Sham* sham-operated RK, *Sham Contra* non-operated contralateral knee

Mice injected i.a. 8 weeks post-DMM surgery displayed an increased retention of Cy5.5-1-11E-scFv in the DMM knee compared with the contralateral knee and sham-operated groups (Fig. 3a; *p* < 0.001). This significant difference was evident as early as 50 hours after i.a. injection where 70.8 ± 9.5 % Cy5.5-1-11E-scFv remained in the DMM-operated joints compared with 32.3 ± 10.8 % in the contralateral knee and 27.9 % ± 13.6 % in the knees that had undergone sham surgery (Fig. 3b; *p* < 0.001). The half-life of Cy5.5-1-11E-scFv in the DMM-operated knee was 81 hours which was over three times longer (*p* < 0.05) than the DMM contralateral (25 hours) and the sham-operated (18 hours) knees. This difference was not seen using Cy5.5-C7-scFv (Fig. 3c; n = 5, *p* = 0.212) with half-lives of 25 hours, 30 hours and 21 hours for DMM, contralateral and sham-operated knee, respectively.

### In vivo optical imaging of Cy5.5-1-11E-mAb in DMM mice following intravenous injection of Cy5.5-1-11E-mAb 4 and 8 weeks post-DMM

**Fig. 4:**
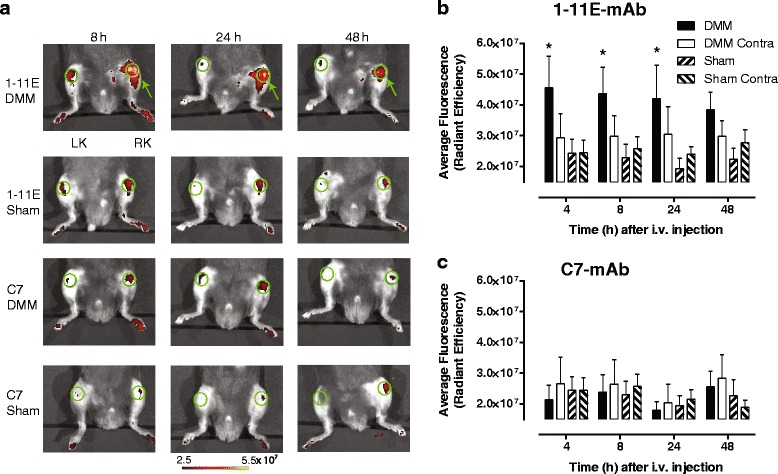
Localization of Cy5.5-1-11E-mAb in mice following i.v injection 4 weeks post-DMM surgery. **a** Representative fluorescent overlay images of mice injected i.v. with 10 μg Cy5.5-1-11E-mAb or Cy5.5-C7-mAb 4 weeks post-surgery. *Green* circles denote the regions of interest used for quantification; *green* arrow indicates the right DMM operated knee (*RK*); *LK* is the non-operated contralateral knee. **b**, **c** Quantification of the amount of antibody detected in the knee joints at the times indicated. Data are mean ± SEM, n = 8. *p < 0.1 by two-way analysis of variance between DMM and sham groups. *DMM* destabilised medial meniscus-operated knee, *DMM Contra* non-operated contralateral knee, *scFv* single chain fragment variable, *Sham* sham-operated RK, *Sham Contra* non-operated contralateral knee

**Fig. 5:**
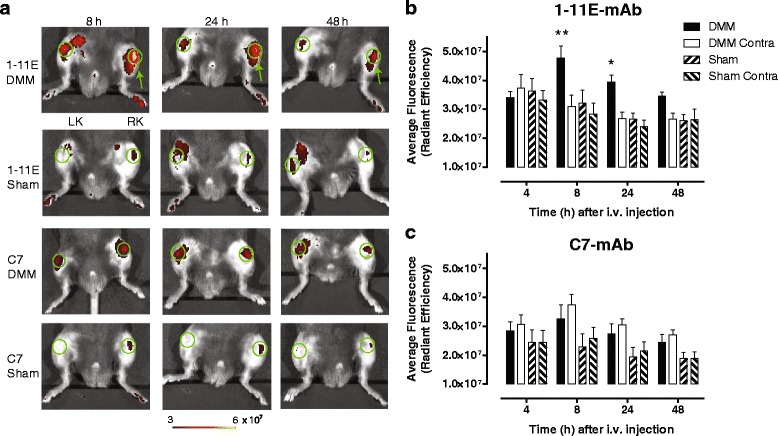
Localization of Cy5.5- 1-11E in mice following i.v injection 8 weeks post-DMM surgery. **a** Representative fluorescent overlay images of mice injected i.v. with 10 μg Cy5.5-1-11E-mAb or Cy5.5-C7-mAb 8 weeks post-surgery. *Green* circles denote the regions of interest used for quantification; *green* arrow indicates the right DMM operated knee (*RK*); *LK* is the non-operated contralateral knee. **b**, **c** Quantification of the amount of antibody detected in the knee joints at the times indicated. Data are mean ± SEM, n = 8. *p < 0.05, **p < 0.01 by two-way analysis of variance between DMM and sham groups. *DMM* destabilised medial meniscus-operated knee, *DMM Contra* non-operated contralateral knee, *scFv* single chain fragment variable, *Sham* sham-operated RK, *Sham Contra* non-operated contralateral knee

Eight animals were injected i.v. with 10 μg Cy5.5-1-11E-mAb or Cy5.5-C7-mAb at 4 weeks and 8 weeks post-DMM and imaged 4, 8, 24 and 48 hours following injection. Representative images are shown from all groups in Fig. 4a (4 weeks post-DMM surgery) and Fig. 5a (8 weeks post-DMM surgery). Quantification of the amount of Cy5.5-1-11E-mAb and Cy5.5-C7-mAb in the knee joint at 4 weeks post-DMM surgery showed significant elevation of 1-11E-mAb localisation in the DMM joint as early as 4 hours post-injection compared with the contralateral knee and joints which had undergone sham surgery (Fig. 4b; *p* = 0.009). This was not observed in Cy5.5-C7-mAb injected animals (Fig. 4c; *p* = 0.911). At 8 weeks post-DMM surgery, there was specific localization of Cy5.5-1-11E-mAb in the DMM joints with maximum signal 8 hours post-Cy5.5-1-11E injection (Fig. 5b; *p* = 0.004), that was not observed in the Cy5.5-C7-mAb injected controls (Fig. 5c; *p* = 0.378).

### Assessment of 1-11E as an imaging biomarker by signal to noise ratio calculation

**Fig. 6:**
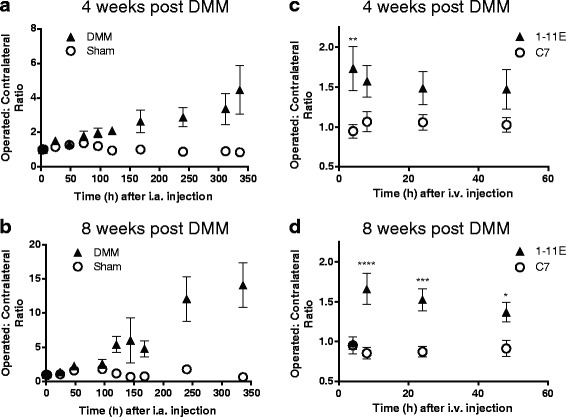
SNRs achieved by 1-11E and C7 full-length antibody and scFv for destabilised medial meniscus (*DMM*) surgery. SNR is expressed as the ratio of signal from the operated knee joint to the corresponding contralateral joint. Cy5.5-1-11E-scFv injected intra-arterially (i.a.) 4 weeks (**a**) and 8 weeks (**b**) post-DMM compared to sham operation (*p* < 0.001 for both 4 and 8 weeks). For mAb, SNR for Cy5.5-1-11E-mAb is compared to Cy5.5-C7-mAb injected intravenously (i.v.) 4 weeks (**c**) and 8 weeks (**d**) post-DMM. *p < 0.05, **p < 0.01, ***p < 0.001, ****p < 0.0001

The above studies demonstrate the validity of 1-11E-scFv and 1-11E-mAb to differentiate OA and sham-operated joints. In order to determine the utility of 1-11E as an imaging biomarker, we calculated the signal to noise ratio (SNR) for each delivery method on an individual animal basis by expressing the signal detected for the operated knee over the contralateral joint. At 4 weeks post-DMM for Cy5.5-1-11E-scFv injected i.a., the SNR was 2.1 ± 0.5 at 120 hours, increasing to 4.5 ± 2.8 at 336 hours, albeit with larger variations (*p* = 0.0014 for SNR of the DMM group versus sham-operated group; Fig. 6a). A larger SNR difference was observed 8 weeks post-DMM surgery where, at 120 hours after i.a. injection, there was 5.4 ± 2.6-times more Cy5.5-1-11E-scFv signal in the DMM-operated knee compared with the contralateral knee. This difference increased at 336 hours after injection to 14.1 ± 4.7 (*p* = 0.0051 for SNR of the DMM group versus sham-operated group; Fig. 6b). The SNR for Cy5.5-1-11E scFv injected i.a. into the sham-operated controls remained at around 1 throughout. The observed SNR for the Cy5.5-1-11E-mAb injected i.v. was very modest at both 4 weeks (Fig. 6c) and 8 weeks (Fig. 6d) post-DMM surgery, with maximum SNR of 1.73 ± 0.78 at 4 hours post-i.v injection and 1.65 ± 0.55 at 8 hours post-injection for 4 and 8 weeks after surgery, respectively. SNR calculated for 1-11E-mAb was significantly higher than the SNR calculated for the group injected with Cy5.5-C7-mAb (*p* < 0.05; Fig. 6c, d) and the sham-operated group (SNR ~ 1; data not shown).

## Discussion

Identification of biomarkers to detect early disease, monitor disease progression, and quantify treatment responses is a priority for OA research as this represents the biggest obstacle to progress for the development of DMOADs. New imaging modalities in combination with genetic and molecular biomarkers will help address this unmet need.

In the current study, we have developed a novel early OA diagnostic imaging tool by combining the use of a molecular biomarker that reflects the metabolic state of chondrocyte at early stages of disease in conjunction with non-invasive imaging. We used a panel of human scFv specific for ROS-CII [21]. Reactive oxygen species (ROS) increase in OA [26, 27] and are thought to result from mitochondrial dysfunction [28] due to ageing (from mitochondrial DNA mutation and oxidative stress [29, 30]) and mechanical stress [31]. The precise role of ROS in the pathogenesis of OA is unclear although they appear to have both beneficial effects, in metabolic adaptation, as well as catabolic effects, e.g. by inducing matrix metalloproteinases (MMPs) [32]. Interestingly, mutations in mitochondrial DNA which accumulate as a result of ROS overproduction correlate with serum markers of cartilage degradation [33, 34].

Following biochemical and functional screening we have selected the clone 1-11E which binds specifically to arthritic cartilage from patients with rheumatoid arthritis and OA as well as mouse cartilage in models of inflammatory arthritis and OA [22]. Staining by 1-11E of the human cartilage did not always correlate with histological features of cartilage degradation, suggesting that this antibody might recognise cartilage alterations prior to histological lesions [21]. We observed territorial and pericellular staining surrounding chondrocyte clusters implying localized high levels of ROS-CII within this region, possibly reflecting enhanced anabolic activity [35]. In the current study, staining of cartilage from DMM-operated knee was evident as early as 4 weeks post-DMM which is prior to significant cartilage degradation. Hence, increased in vivo signal was evident as early as 4 weeks post-DMM compared with either sham operation or a negative control antibody. Moreover, the difference between controls and DMM knees increased over time and cartilage degradation. Collectively, these results suggest that 1-11E is a specific early diagnostic biomarker and could potentially measure the degree of cartilage damage and response to treatment(s).

We examined targeting ROS-CII using two alternative antibody formats: Cy5.5-1-11E-scFv and Cy5.5-1-11E-mAb. Although both approaches demonstrated similar sensitivity in in-vivo imaging (first detected significant increased signal at 4 weeks post-DMM), their molecular weight differences (25 kDa vs 150 kDa, respectively) results in different pharmacokinetics. Delivery of Cy5.5-1-11E-scFv required i.a. injection as no specific retention was observed following i.v. administration (data not shown). This was most likely due to its rapid systemic clearance (blood half-life of scFv is 2 hours [36]) and relatively slow extravasation into the OA joint, assuming no change in synovial permeability during disease progression. To overcome this problem we designed the intact mAb (blood half-life of mAb is 110 hours [36]) to reduce its systemic clearance rate. Indeed Cy5.5-1-11E-mAb injected i.v. localised specifically in DMM knees compared with the contralateral knee and sham-operated knees as early as 4 weeks post-DMM. These results demonstrate that systemic delivery of an antibody targeting ROS-CII is possible although with lower sensitivity (as shown by SNR analysis) compared with i.a. delivery.

The higher SNR of the i.a. delivered scFv compared to the i.v. delivered mAb could be attributed to both the higher local concentration of scFv and the lower background signal due to the absence of circulating scFv, especially after 100 hours post-i.a. injection. The SNR of the i.v. delivered mAb could possibly be increased by increasing the dose of mAb.

In summary, systemic or local delivery of 1-11E is able to target the OA joint specifically and may represent an early biomarker of OA ahead of radiographic changes. It remains to be seen whether this biomarker could also be used to monitor disease progression, which could infer important utility in preclinical drug screening and clinical trials. These have important impacts for refining and reducing animal use in research according to the principals of the 3Rs (see www.nc3rs.org.uk). Future imaging study will be needed for further optimisation of 1-11E-mAb imaging following systemic administration. In addition, 1-11E coupled with the other optical probes, such as the MMP-activated probes, may further increase sensitivity.

Alternative efforts have largely focussed on developing MRI technologies to detect early abnormalities at the pre-radiographic stage [37, 38]. For small animals, equilibrium partitioning of an ionic contrast-microcomputed tomography [39] and phosphotungstic acid [40] provide excellent cartilage/bone contrast for volumetric evaluation by microcomputed tomography although they are yet suitable for in vivo use.

Other strategies for early imaging include approaches that target pathogenic activities within the joint to directly examine the metabolic state and the microenvironment during the development of OA in real time. Cleavable peptide probes that are specific for MMPs have been used experimentally to visualise catabolic activity within the joint providing sensitive and consistent visualisation of OA progression. Using MMP-generic [41, 42] or MMP-13-specific [43] probes, imaging showed significantly higher fluorescence intensity in OA knees compared to sham-operated knees of the same mice.

## Conclusions

In patients, radiographic biomarkers have potential advantages over circulating ones as they provide information at a given joint site and are not influenced by systemic factors, e.g. inflammation, OA at other sites or liver/kidney dysfunction. Near-infrared fluorescence imaging used in the current study is a potential imaging modality for small human joints. However, optical imaging has limited spatial resolution and tissue penetration [33, 44] and development of 1-11E for positron emission tomography and single positron emission computed tomography will be required for larger joint imaging. Looking towards the future, the long retention time of the 1-11E-scFv in the OA joint or the specific localization of 1-11E-mAb to the joint with OA may exploit the development of 1-11E for pharmacodelivery of agents to focal areas of disease thus improving the efficacy and safety profile of new DMOADs.

## Abbreviations

DMM: Destabilised medial meniscus
DMOAD: Disease-modifying osteoathritis drug
i.a.: Intra-articular
i.v.: Intravenous
mAb: Monoclonal antibody
MMP: Matrix metalloproteinase
MRI: Magnetic resonance imaging
OA: Osteoarthritis
ROS: Reactive oxygen species
ROS-CII: Collagen type II post-translationally modified by reactive oxidant species
scFv: Single chain fragment variable
SNR: signal to noise ratio
